# Tetra­aqua­bis­(2,3-di­hydro-1,4-benzodioxine-2-carboxyl­ato)calcium(II)

**DOI:** 10.1107/S2414314620010925

**Published:** 2020-08-14

**Authors:** Esmit B. Camargo-Cortés, Mirna Acosta, Juan C. Martínez, Leslie W. Pineda

**Affiliations:** a Centro Especializado en Investigaciones en Química Inorgánica (CEIQUI), Escuela de Química, Universidad Autónoma de Chiriquí, David, Panama; b Centro de Electroquímica y Energía Química (CELEQ), Universidad de Costa Rica, 11501-2060. San José, Costa Rica; c Escuela de Química, Universidad de Costa Rica, 11501-2060, San José, Costa Rica; University of Aberdeen, Scotland

**Keywords:** crystal structure, 1,4-benzodioxane, calcium atom, carboxyl­ate groups, hydrogen bonding

## Abstract

The acid–base reaction of 1,4-benzodioxane 2-carb­oxy­lic acid with calcium carbonate furnished the centrosymmetric title compound, [Ca(C_9_H_7_O_4_)_2_(H_2_O)_4_], in which the metal ion is octa­hedrally coordinated by two monodentate 1,4-benzodioxane 2-carboxyl­ate ligands and four water mol­ecules. In the crystal, O—H⋯O and C—H⋯O hydrogen bonds link the mol­ecules into a three-dimensional network.

## Abstract 2

In the title coordination compound, the metal ion is bonded to two monodentate carboxyl­ate groups and four water mol­ecules.

## Graphical abstract

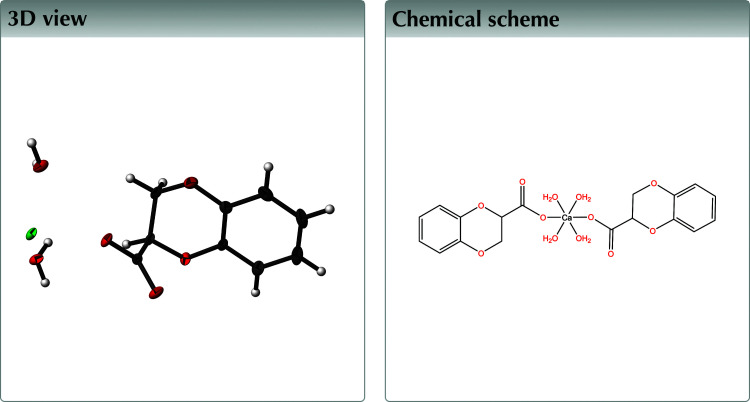

## Structure description

1,4-Benzodioxanes are components of some therapeutic agents used in cardiovascular treatments, acting as α- and β-adrenergic antagonists (Nelson *et al.*, 1977 ▸, 1979 ▸; Pigini *et al.*, 1988 ▸). For the latter application, the enanti­opure derivatives of chiral 2-substituted 1,4-benzodioxanes lend affinity and selectivity, mainly those derived from 1,4-benzodioxane 2-carb­oxy­lic acid (Ennis & Old, 1992 ▸; Antus *et al.*, 1993 ▸; Khouili *et al.*, 1999 ▸; Jasinski *et al.*, 2009 ▸). Naturally ocurring compounds with a similar structure to these heterocyclic scaffolds are known as 1,4-benzodioxane lignans, which also exhibit a wide array of biological activities (*e.g.*, anti­cancer, anti­oxidant; Pilkington & Barker, 2015 ▸).

In this work, we report the synthesis and the structure of the coordination properties of 1,4-benzodioxane 2-carb­oxy­lic acid toward calcium carbonate to afford the title compound Ca(C_9_H_7_O_4_)_2_(H_2_O)_4_.

**Figure 1:**
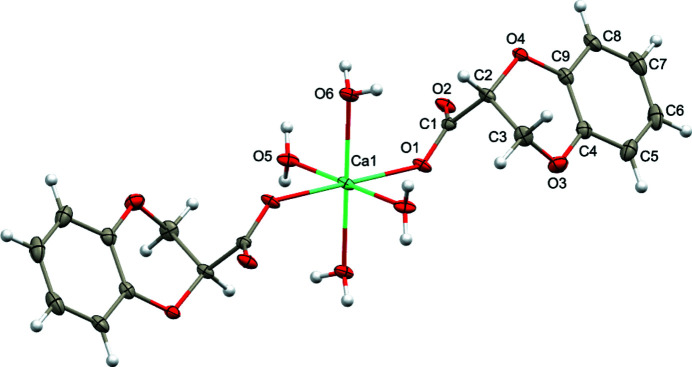
The mol­ecular structure of the title compound with displacement ellipsoids drawn at the 50% probability level. Unlabeled atoms are generated by the symmetry operation 1 − *x*, 1 − *y*, 2 − *z*.

The crystal structure of the title compound has monoclinic symmetry with half a mol­ecule in the asymmetric unit, the other half being generated by a crystallographic inversion center. The calcium ion is bonded to four aqua ligands and two 1,4-benzodioxane 2-carboxyl­ate ligands, whose carboxyl­ate groups link to the central atom in monodentate mode (Fig. 1 ▸). The Ca1—O1, Ca1—O5, and Ca1—O6 bond lengths are 2.304 (2), 2.358 (2) and 2.317 (2) Å, respectively. The dioxane ring adopts a half-chair conformation with the pendant carboxyl­ate group in an axial orientation. In the arbitrarily chosen asymmetric unit, C2 has an *R* configuration but crystal symmetry generates a racemic mixture.

**Figure 2:**
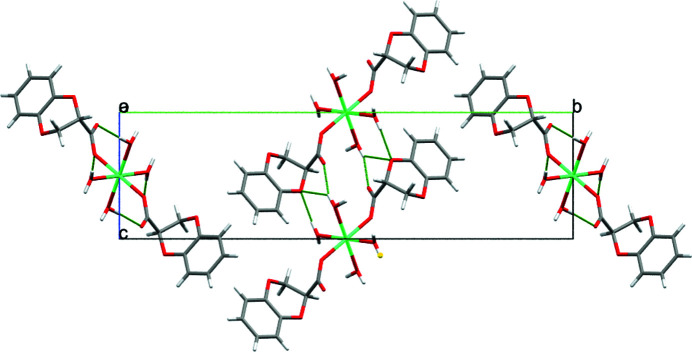
Packing of the mol­ecules of the title compound. O—H⋯O and C—H⋯O hydrogen bonds are shown as green dashed lines.

In the crystal, O—H⋯O hydrogen bonds link the mol­ecules into (010) sheets with the acceptor O atoms being parts of carboxyl­ate groups (O1 and O2) and the dioxane ring (O4) and the packing is consolidated by weak C—H⋯O inter­actions (Fig. 2 ▸, Table 1 ▸).

## Synthesis and crystallization

In a 100 mL two-necked flask, anhydrous CaCO_3_ (0.0100 g, 0.100 mmol) was dissolved in deionized water (20 mL) by heating to 338 K, and a solution of 1,4-benzodioxane-2-carb­oxy­lic acid (0.0560 g, 0.200 mmol) dissolved in distilled water (10 mL) was added dropwise at 353 K. The reaction mixture was refluxed for 2 h and then concentrated under vacuum to 10 mL. The precipitate obtained upon cooling overnight was filtered off and washed with cold distilled water. Colorless crystals suitable for X-ray analysis were grown from a warm water–methanol mixed solvent mixture (1:1) at room temperature. Yield: 0.0362 g (55%), m.p. 501–505 K. FTIR data (KBr, cm^−1^): 3600 and 3000 (*br*, *m*); 3568 (*m*); 1330 (*m*); 879 (*m*); 833 (*s*); 767 (*s*); 752 (*s*); 654 (*m*); 569 (*m*); 545 (*w*); 476 (*m*). ^1^H NMR (400 MHz, mix 1:1 D_2_O: CD_4_O, 298 K): *δ* 6.87–6.99 (*s*, 4H), 4.82 (*dd*, 1H), 4.35 p.p.m. (*qd*, 2H). ^13^C NMR (400 MHz, 1:1 mix D_2_O: CD_4_O, 298 K): *δ* 176, 143, 142, 123, 122, 117, 115, 73.5, 66 p.p.m. The ^1^H NMR and ^13^C NMR spectra for the title compound are included in the supporting information.

## Refinement

Crystal data, data collection and structure refinement details are summarized in Table 2 ▸.

## Supplementary Material

Crystal structure: contains datablock(s) global, I. DOI: 10.1107/S2414314620010925/hb4355sup1.cif

Structure factors: contains datablock(s) I. DOI: 10.1107/S2414314620010925/hb4355Isup2.hkl

1H and 13C NMR spectra of the title compound. DOI: 10.1107/S2414314620010925/hb4355sup3.pdf

CCDC reference: 1970573

Additional supporting information:  crystallographic information; 3D view; checkCIF report

## Figures and Tables

**Table 1:** Hydrogen-bond geometry (Å, °)

*D*—H⋯*A*	*D*—H	H⋯*A*	*D*⋯*A*	*D*—H⋯*A*
O5—H5*A*⋯O1^i^	0.84 (2)	1.96 (2)	2.800 (3)	178 (4)
O5—H5*B*⋯O4^ii^	0.84 (3)	2.07 (3)	2.828 (3)	149 (4)
O6—H6*A*⋯O2^iii^	0.83 (3)	1.93 (2)	2.707 (3)	155 (3)
O6—H6*B*⋯O2^i^	0.84 (3)	1.88 (4)	2.715 (3)	176 (3)
C2—H2⋯O2^i^	1.00	2.53	3.379 (4)	143
C6—H6⋯O3^iv^	0.95	2.54	3.357 (4)	145

Symmetry codes: (i) 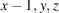 ; (ii) 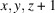 ; (iii) 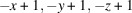 ; (iv) 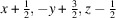 .

**Table 2:** Experimental details

Crystal data	
Chemical formula	[Ca(C_9_H_7_O_4_)_2_(H_2_O)_4_]
*M*_r_	470.44
Crystal system, space group	Monoclinic, *P*2_1_/*n*
Temperature (K)	100
*a*, *b*, *c* (Å)	5.3477 (4), 26.6084 (18), 7.7367 (5)
β (°)	106.715 (2)
*V* (Å^3^)	1054.37 (13)
*Z*	2
Radiation type	Mo *K*α
μ (mm^−1^)	0.36
Crystal size (mm)	0.35 × 0.25 × 0.15

Data collection	
Diffractometer	Bruker D8 Venture
Absorption correction	Multi-scan (*SADABS*; Bruker, 2015 ▸)
*T*_min_, *T*_max_	0.661, 0.746
No. of measured, independent and observed [*I* > 2σ(*I*)] reflections	32373, 2384, 2242
*R*_int_	0.036
(sin θ/λ)_max_ (Å^−1^)	0.647

Refinement	
*R*[*F* ^2^ > 2σ(*F* ^2^)], *wR*(*F* ^2^), *S*	0.057, 0.133, 1.39
No. of reflections	2384
No. of parameters	158
No. of restraints	6
H-atom treatment	H atoms treated by a mixture of independent and constrained refinement
Δρ_max_, Δρ_min_ (e Å^−3^)	0.42, −0.43

Computer programs: *APEX3* and *SAINT* (Bruker, 2015 ▸), *SHELXT* (Sheldrick, 2015*a* ▸), *SHELXL2014* (Sheldrick, 2015*b* ▸), *Mercury* (Macrae *et al.*, 2020 ▸) and *publCIF* (Westrip, 2010 ▸).

## full crystallographic data

### Crystal data

**Table:**

[Ca(C_9_H_7_O_4_)_2_(H_2_O)_4_]	*F*(000) = 492
*M**_r_* = 470.44	*D*_x_ = 1.482 Mg m^−^^3^
Monoclinic, *P*2_1_/*n*	Mo *K*α radiation, λ = 0.71073 Å
*a* = 5.3477 (4) Å	Cell parameters from 9843 reflections
*b* = 26.6084 (18) Å	θ = 2.9–27.4°
*c* = 7.7367 (5) Å	µ = 0.36 mm^−^^1^
β = 106.715 (2)°	*T* = 100 K
*V* = 1054.37 (13) Å^3^	Block, clear light white
*Z* = 2	0.35 × 0.25 × 0.15 mm

### Data collection

**Table:**

Bruker D8 Venture diffractometer	2384 independent reflections
Mirrors monochromator	2242 reflections with *I* > 2σ(*I*)
Detector resolution: 10.4167 pixels mm^-1^	*R*_int_ = 0.036
ω scans	θ_max_ = 27.4°, θ_min_ = 2.9°
Absorption correction: multi-scan (SADABS; Bruker, 2015)	*h* = −6→6
*T*_min_ = 0.661, *T*_max_ = 0.746	*k* = −34→34
32373 measured reflections	*l* = −10→8

### Refinement

**Table:**

Refinement on *F*^2^	Primary atom site location: structure-invariant direct methods
Least-squares matrix: full	Secondary atom site location: difference Fourier map
*R*[*F*^2^ > 2σ(*F*^2^)] = 0.057	Hydrogen site location: mixed
*wR*(*F*^2^) = 0.133	H atoms treated by a mixture of independent and constrained refinement
*S* = 1.39	*w* = 1/[σ^2^(*F*_o_^2^) + 3.3804*P*] where *P* = (*F*_o_^2^ + 2*F*_c_^2^)/3
2384 reflections	(Δ/σ)_max_ < 0.001
158 parameters	Δρ_max_ = 0.42 e Å^−^^3^
6 restraints	Δρ_min_ = −0.43 e Å^−^^3^

### Special details

**Table:**

Geometry. All esds (except the esd in the dihedral angle between two l.s. planes) are estimated using the full covariance matrix. The cell esds are taken into account individually in the estimation of esds in distances, angles and torsion angles; correlations between esds in cell parameters are only used when they are defined by crystal symmetry. An approximate (isotropic) treatment of cell esds is used for estimating esds involving l.s. planes.

### Fractional atomic coordinates and isotropic or equivalent isotropic displacement parameters (Å^2^)

**Table:**

	*x*	*y*	*z*	*U*_iso_*/*U*_eq_	
Ca1	0.5	0.5	1.0	0.0115 (2)	
O1	0.6722 (4)	0.55435 (8)	0.8323 (3)	0.0144 (4)	
O2	0.8347 (4)	0.54948 (9)	0.5982 (3)	0.0164 (5)	
O3	0.5527 (5)	0.66992 (9)	0.6702 (3)	0.0199 (5)	
O4	0.4402 (4)	0.60021 (8)	0.3776 (3)	0.0144 (4)	
O5	0.2000 (4)	0.56223 (9)	1.0277 (3)	0.0167 (5)	
H5A	0.041 (3)	0.5599 (16)	0.972 (4)	0.030 (12)*	
H5B	0.210 (7)	0.5750 (15)	1.129 (3)	0.033 (12)*	
O6	0.1782 (4)	0.47510 (9)	0.7438 (3)	0.0158 (5)	
H6A	0.208 (8)	0.4613 (13)	0.655 (3)	0.028 (12)*	
H6B	0.070 (7)	0.4981 (12)	0.703 (5)	0.043 (14)*	
C1	0.6624 (6)	0.56196 (11)	0.6687 (4)	0.0116 (6)	
C2	0.4144 (6)	0.58769 (12)	0.5516 (4)	0.0121 (6)	
H2	0.2671	0.5632	0.5335	0.015*	
C3	0.3443 (6)	0.63398 (12)	0.6413 (5)	0.0171 (6)	
H3A	0.1807	0.6488	0.5636	0.021*	
H3B	0.316	0.6247	0.7582	0.021*	
C4	0.6346 (6)	0.67786 (12)	0.5198 (4)	0.0160 (6)	
C5	0.7822 (7)	0.72053 (13)	0.5151 (5)	0.0226 (7)	
H5	0.8188	0.7437	0.6124	0.027*	
C6	0.8762 (7)	0.72926 (13)	0.3682 (5)	0.0261 (8)	
H6	0.9784	0.7583	0.3656	0.031*	
C7	0.8210 (7)	0.69567 (14)	0.2255 (5)	0.0251 (8)	
H7	0.8838	0.702	0.1246	0.03*	
C8	0.6745 (6)	0.65272 (13)	0.2289 (4)	0.0191 (7)	
H8	0.6373	0.6297	0.1311	0.023*	
C9	0.5828 (6)	0.64388 (11)	0.3781 (4)	0.0129 (6)	

### Atomic displacement parameters (Å^2^)

**Table:**

	*U*^11^	*U*^22^	*U*^33^	*U*^12^	*U*^13^	*U*^23^
Ca1	0.0075 (4)	0.0198 (4)	0.0070 (4)	0.0002 (3)	0.0018 (3)	0.0016 (3)
O1	0.0118 (10)	0.0243 (12)	0.0078 (10)	0.0001 (8)	0.0037 (8)	0.0026 (8)
O2	0.0128 (10)	0.0270 (12)	0.0100 (10)	0.0058 (9)	0.0041 (8)	0.0011 (9)
O3	0.0215 (12)	0.0206 (12)	0.0194 (12)	−0.0010 (9)	0.0089 (9)	−0.0046 (9)
O4	0.0146 (10)	0.0181 (11)	0.0091 (10)	−0.0028 (8)	0.0013 (8)	0.0012 (8)
O5	0.0087 (10)	0.0279 (12)	0.0125 (11)	0.0006 (9)	0.0015 (8)	−0.0042 (9)
O6	0.0144 (11)	0.0227 (12)	0.0095 (10)	0.0042 (9)	0.0022 (8)	−0.0015 (9)
C1	0.0105 (13)	0.0133 (13)	0.0107 (13)	−0.0024 (10)	0.0025 (11)	−0.0008 (10)
C2	0.0080 (13)	0.0196 (15)	0.0090 (13)	−0.0004 (11)	0.0027 (10)	0.0020 (11)
C3	0.0113 (14)	0.0227 (16)	0.0209 (16)	0.0027 (12)	0.0103 (12)	0.0029 (13)
C4	0.0125 (14)	0.0164 (15)	0.0179 (15)	0.0035 (11)	0.0023 (12)	0.0026 (12)
C5	0.0173 (16)	0.0154 (15)	0.0330 (19)	0.0005 (12)	0.0039 (14)	0.0005 (14)
C6	0.0172 (17)	0.0210 (17)	0.038 (2)	−0.0025 (13)	0.0051 (15)	0.0121 (15)
C7	0.0187 (16)	0.0316 (19)	0.0244 (18)	0.0005 (14)	0.0053 (14)	0.0144 (15)
C8	0.0182 (16)	0.0249 (17)	0.0124 (15)	0.0016 (13)	0.0016 (12)	0.0059 (12)
C9	0.0077 (13)	0.0177 (15)	0.0141 (14)	0.0025 (11)	0.0041 (11)	0.0042 (11)

### Geometric parameters (Å, º)

**Table:**

Ca1—O1^i^	2.304 (2)	O6—H6B	0.839 (10)
Ca1—O1	2.304 (2)	C1—C2	1.535 (4)
Ca1—O6^i^	2.317 (2)	C2—C3	1.513 (4)
Ca1—O6	2.317 (2)	C2—H2	1.0
Ca1—O5^i^	2.358 (2)	C3—H3A	0.99
Ca1—O5	2.358 (2)	C3—H3B	0.99
Ca1—H6B	2.74 (4)	C4—C9	1.386 (4)
O1—C1	1.268 (4)	C4—C5	1.389 (5)
O2—C1	1.243 (4)	C5—C6	1.388 (5)
O3—C4	1.372 (4)	C5—H5	0.95
O3—C3	1.437 (4)	C6—C7	1.385 (6)
O4—C9	1.389 (4)	C6—H6	0.95
O4—C2	1.432 (3)	C7—C8	1.390 (5)
O5—H5A	0.838 (10)	C7—H7	0.95
O5—H5B	0.838 (10)	C8—C9	1.397 (4)
O6—H6A	0.837 (10)	C8—H8	0.95
			
O1^i^—Ca1—O1	180.0	O1—C1—C2	116.1 (3)
O1^i^—Ca1—O6^i^	90.95 (8)	O4—C2—C3	110.2 (2)
O1—Ca1—O6^i^	89.05 (8)	O4—C2—C1	111.0 (2)
O1^i^—Ca1—O6	89.05 (8)	C3—C2—C1	112.3 (2)
O1—Ca1—O6	90.95 (8)	O4—C2—H2	107.7
O6^i^—Ca1—O6	180.00 (10)	C3—C2—H2	107.7
O1^i^—Ca1—O5^i^	90.17 (8)	C1—C2—H2	107.7
O1—Ca1—O5^i^	89.83 (8)	O3—C3—C2	109.3 (2)
O6^i^—Ca1—O5^i^	85.49 (8)	O3—C3—H3A	109.8
O6—Ca1—O5^i^	94.51 (8)	C2—C3—H3A	109.8
O1^i^—Ca1—O5	89.83 (8)	O3—C3—H3B	109.8
O1—Ca1—O5	90.17 (8)	C2—C3—H3B	109.8
O6^i^—Ca1—O5	94.51 (8)	H3A—C3—H3B	108.3
O6—Ca1—O5	85.49 (8)	O3—C4—C9	122.0 (3)
O5^i^—Ca1—O5	180.0	O3—C4—C5	118.1 (3)
O1^i^—Ca1—H6B	94.9 (10)	C9—C4—C5	119.9 (3)
O1—Ca1—H6B	85.1 (9)	C6—C5—C4	120.0 (3)
O6^i^—Ca1—H6B	163.5 (6)	C6—C5—H5	120.0
O6—Ca1—H6B	16.5 (6)	C4—C5—H5	120.0
O5^i^—Ca1—H6B	109.8 (6)	C7—C6—C5	120.0 (3)
O5—Ca1—H6B	70.2 (6)	C7—C6—H6	120.0
C1—O1—Ca1	138.9 (2)	C5—C6—H6	120.0
C4—O3—C3	113.1 (2)	C6—C7—C8	120.5 (3)
C9—O4—C2	113.2 (2)	C6—C7—H7	119.7
Ca1—O5—H5A	121 (3)	C8—C7—H7	119.7
Ca1—O5—H5B	120 (3)	C7—C8—C9	119.2 (3)
H5A—O5—H5B	107 (2)	C7—C8—H8	120.4
Ca1—O6—H6A	124 (3)	C9—C8—H8	120.4
Ca1—O6—H6B	112 (3)	C4—C9—O4	122.1 (3)
H6A—O6—H6B	106 (2)	C4—C9—C8	120.4 (3)
O2—C1—O1	124.9 (3)	O4—C9—C8	117.5 (3)
O2—C1—C2	118.9 (3)		
			
Ca1—O1—C1—O2	−102.2 (4)	O3—C4—C5—C6	178.0 (3)
Ca1—O1—C1—C2	76.5 (4)	C9—C4—C5—C6	0.4 (5)
C9—O4—C2—C3	45.0 (3)	C4—C5—C6—C7	0.6 (5)
C9—O4—C2—C1	−79.9 (3)	C5—C6—C7—C8	−0.8 (5)
O2—C1—C2—O4	−8.8 (4)	C6—C7—C8—C9	0.2 (5)
O1—C1—C2—O4	172.4 (2)	O3—C4—C9—O4	1.4 (5)
O2—C1—C2—C3	−132.6 (3)	C5—C4—C9—O4	178.9 (3)
O1—C1—C2—C3	48.5 (4)	O3—C4—C9—C8	−178.6 (3)
C4—O3—C3—C2	48.5 (3)	C5—C4—C9—C8	−1.1 (5)
O4—C2—C3—O3	−63.0 (3)	C2—O4—C9—C4	−15.5 (4)
C1—C2—C3—O3	61.3 (3)	C2—O4—C9—C8	164.4 (3)
C3—O3—C4—C9	−19.4 (4)	C7—C8—C9—C4	0.8 (5)
C3—O3—C4—C5	163.1 (3)	C7—C8—C9—O4	−179.1 (3)

Symmetry code: (i) −*x*+1, −*y*+1, −*z*+2.

### Hydrogen-bond geometry (Å, º)

**Table:**

*D*—H···*A*	*D*—H	H···*A*	*D*···*A*	*D*—H···*A*
O5—H5*A*···O1^ii^	0.84 (2)	1.96 (2)	2.800 (3)	178 (4)
O5—H5*B*···O4^iii^	0.84 (3)	2.07 (3)	2.828 (3)	149 (4)
O6—H6*A*···O2^iv^	0.83 (3)	1.93 (2)	2.707 (3)	155 (3)
O6—H6*B*···O2^ii^	0.84 (3)	1.88 (4)	2.715 (3)	176 (3)
C2—H2···O2^ii^	1.00	2.53	3.379 (4)	143
C6—H6···O3^v^	0.95	2.54	3.357 (4)	145

Symmetry codes: (ii) *x*−1, *y*, *z*; (iii) *x*, *y*, *z*+1; (iv) −*x*+1, −*y*+1, −*z*+1; (v) *x*+1/2, −*y*+3/2, *z*−1/2.
