# Supplementary material for: Tetra­aqua­bis­(2,3-di­hydro-1,4-benzodioxine-2-carboxyl­ato)calcium(II)
Source: IUCrdata. 2020 Aug 14;5(Pt 8):x201092. doi: 10.1107/S2414314620010925 (PMC9462237; doi:10.1107/S2414314620010925)
Supplement: Supplementary file 3 [file x-05-x201092-sup3.pdf]

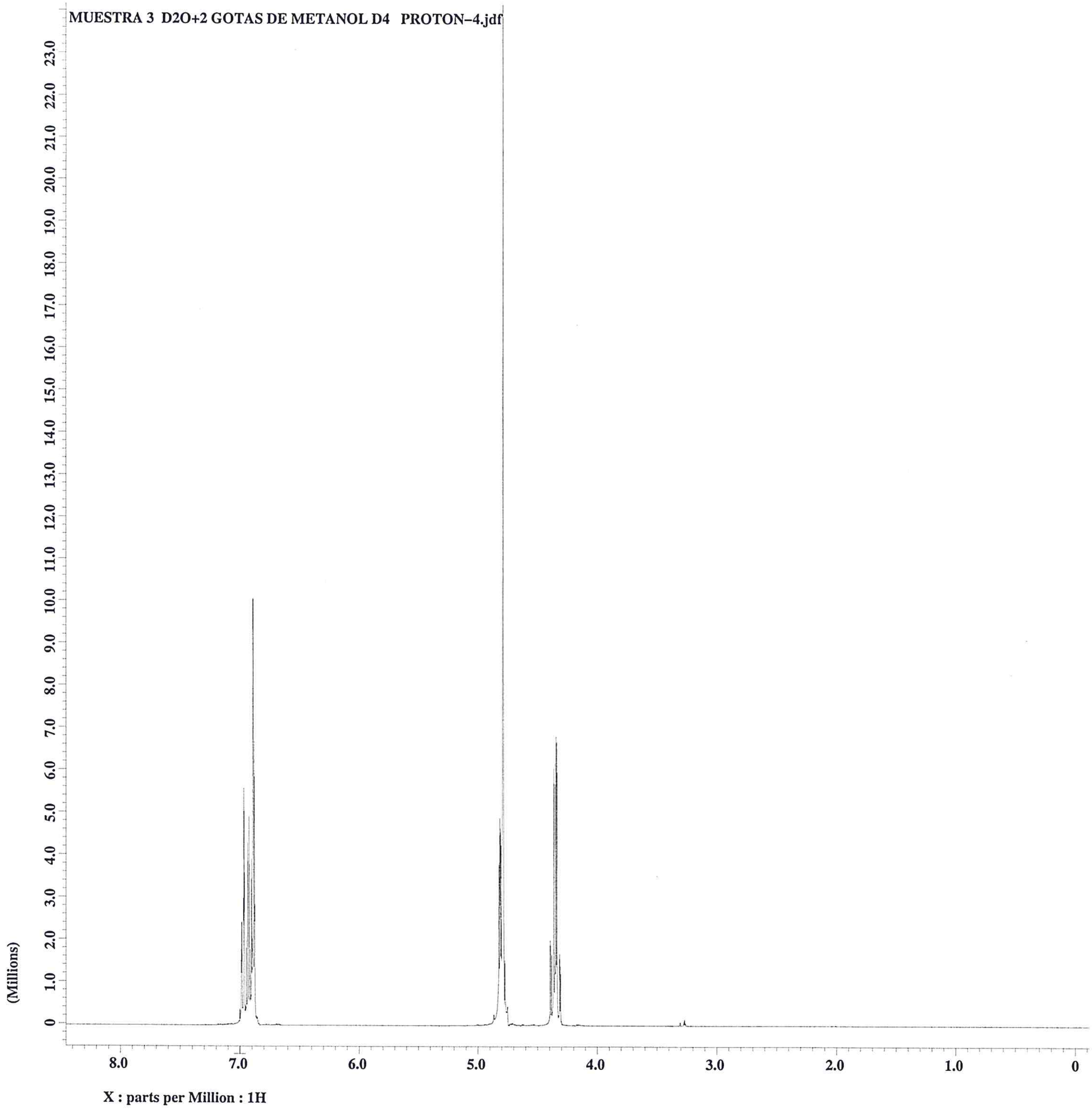


| | |
|---|---|
| Filename | = MUESTRA 3 D2O+2 GOTA |
| Experiment | = single_pulse.exp |
| Sample_id | = I3_030_009 |
| Solvent | = D2O |
| Creation_time | = 3-DEC-2014 14:05:36 |
| Author | = DELTA |
| Content | = Single Pulse Experime |
| Current_time | = 4-DEC-2014 12:48:47 |
| Data_format | = 1D COMPLEX |
| Dim_size | = 16384 |
| Dim_title | = 1H |
| Dim_units | = [ppm] |
| Dimensions | = X |
| Machine | = eclipse2 |
| Revision_time | = 3-DEC-2014 14:56:13 |
| Site | = Eclipse+ 400 |
| Spectrometer | = DELTA_NMR |
| Field_strength | = 9.389766[T] (400[MHz] |
| X_acq_duration | = 2.048[s] |
| X_domain | = 1H |
| X_freq | = 399.78219838[MHz] |
| X_offset | = 7[ppm] |
| X_points | = 16384 |
| X_prescans | = 0 |
| X_resolution | = 0.48828125[Hz] |
| X_sweep | = 8[kHz] |
| Clipped | = FALSE |
| Mod_return | = 1 |
| Scans | = 24 |
| Total_scans | = 24 |
| X_90_width | = 9.74[us] |
| X_acq_time | = 2.048[s] |
| X_angle | = 45[deg] |
| X_pulse | = 4.87[us] |
| Initial_wait | = 1[s] |
| Phase_preset | = 3[us] |
| Recvr_gain | = 12 |
| Relaxation_delay | = 4[s] |
| Temp_get | = 22.1[dC] |
| Unblank_time | = 2[us] |

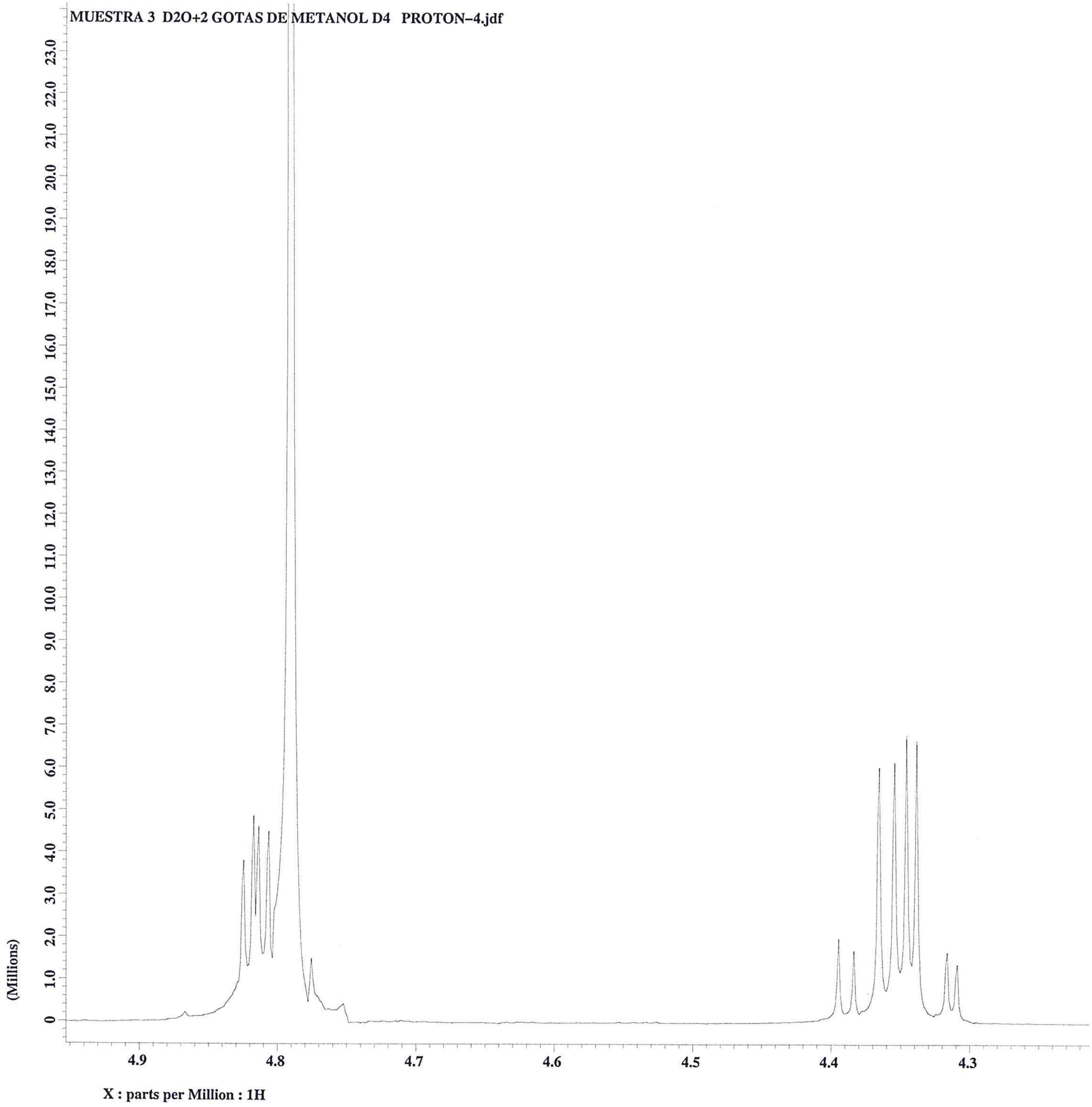


| | |
|---|---|
| Filename | = MUESTRA 3 D2O+2 GOTA |
| Experiment | = single_pulse.exp |
| Sample_id | = I3_030_009 |
| Solvent | = D2O |
| Creation_time | = 3-DEC-2014 14:05:36 |
| Author | = DELTA |
| Content | = Single Pulse Experime |
| Current_time | = 4-DEC-2014 12:48:59 |
| Data_format | = 1D COMPLEX |
| Dim_size | = 16384 |
| Dim_title | = 1H |
| Dim_units | = [ppm] |
| Dimensions | = X |
| Machine | = eclipse2 |
| Revision_time | = 3-DEC-2014 14:56:13 |
| Site | = Eclipse+ 400 |
| Spectrometer | = DELTA_NMR |
| Field_strength | = 9.389766[T] (400[MHz] |
| X_acq_duration | = 2.048[s] |
| X_domain | = 1H |
| X_freq | = 399.78219838[MHz] |
| X_offset | = 7[ppm] |
| X_points | = 16384 |
| X_prescans | = 0 |
| X_resolution | = 0.48828125[Hz] |
| X_sweep | = 8[kHz] |
| Clipped | = FALSE |
| Mod_return | = 1 |
| Scans | = 24 |
| Total_scans | = 24 |
| X_90_width | = 9.74[us] |
| X_acq_time | = 2.048[s] |
| X_angle | = 45[deg] |
| X_pulse | = 4.87[us] |
| Initial_wait | = 1[s] |
| Phase_preset | = 3[us] |
| Recvr_gain | = 12 |
| Relaxation_delay | = 4[s] |
| Temp_get | = 22.1[dC] |
| Unblank_time | = 2[us] |

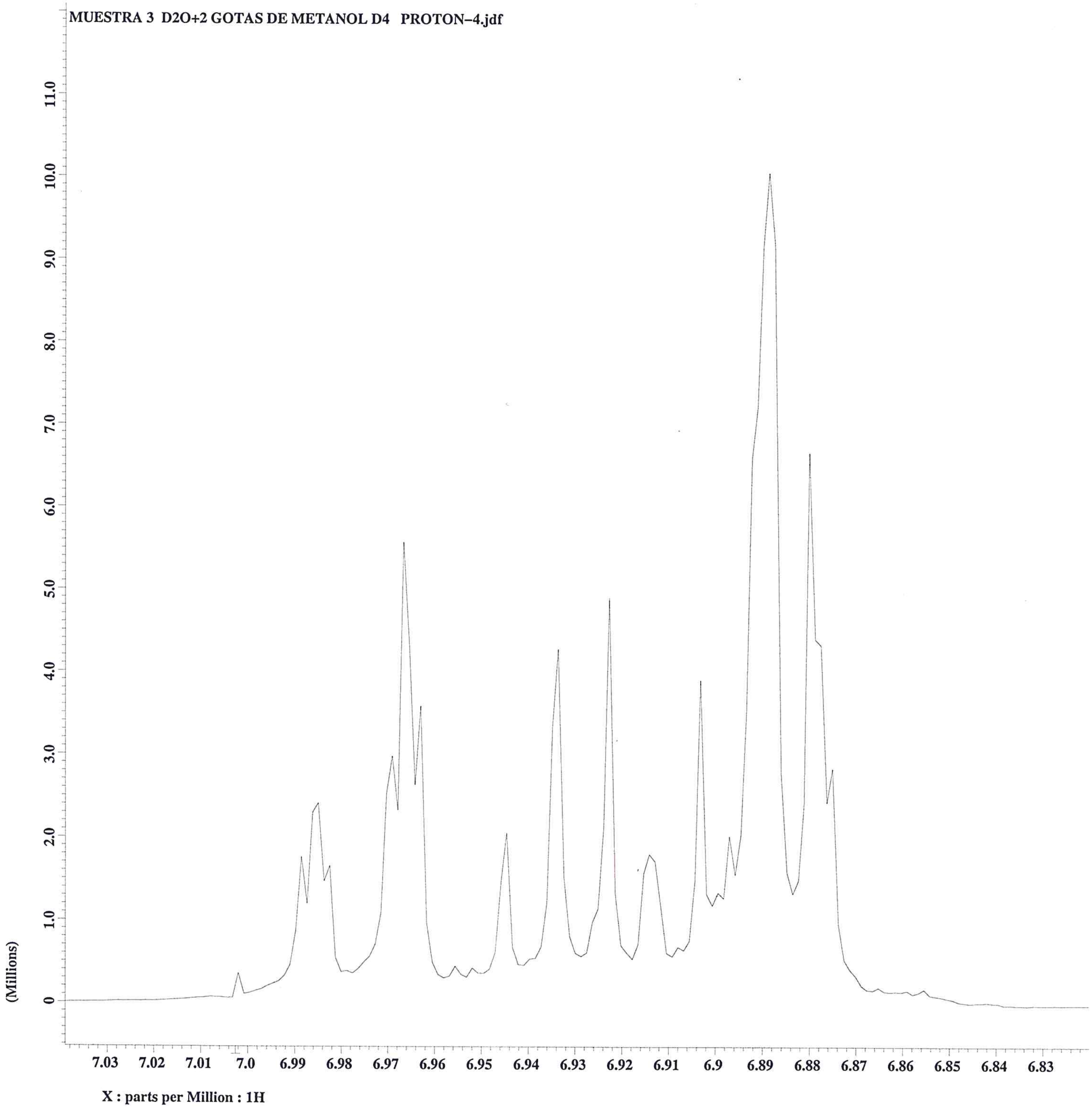


| | |
|---|---|
| Filename | = MUESTRA 3 D2O+2 GOTA |
| Experiment | = single_pulse.exp |
| Sample_id | = I3_030_009 |
| Solvent | = D2O |
| Creation_time | = 3-DEC-2014 14:05:36 |
| Author | = DELTA |
| Content | = Single Pulse Experime |
| Current_time | = 4-DEC-2014 12:49:24 |
| Data_format | = 1D COMPLEX |
| Dim_size | = 16384 |
| Dim_title | = 1H |
| Dim_units | = [ppm] |
| Dimensions | = X |
| Machine | = eclipse2 |
| Revision_time | = 3-DEC-2014 14:56:13 |
| Site | = Eclipse+ 400 |
| Spectrometer | = DELTA_NMR |
| Field_strength | = 9.389766[T] (400[MHz] |
| X_acq_duration | = 2.048[s] |
| X_domain | = 1H |
| X_freq | = 399.78219838[MHz] |
| X_offset | = 7[ppm] |
| X_points | = 16384 |
| X_prescans | = 0 |
| X_resolution | = 0.48828125[Hz] |
| X_sweep | = 8[kHz] |
| Clipped | = FALSE |
| Mod_return | = 1 |
| Scans | = 24 |
| Total_scans | = 24 |
| X_90_width | = 9.74[us] |
| X_acq_time | = 2.048[s] |
| X_angle | = 45[deg] |
| X_pulse | = 4.87[us] |
| Initial_wait | = 1[s] |
| Phase_preset | = 3[us] |
| Recvr_gain | = 12 |
| Relaxation_delay | = 4[s] |
| Temp_get | = 22.1[dC] |
| Unblank_time | = 2[us] |

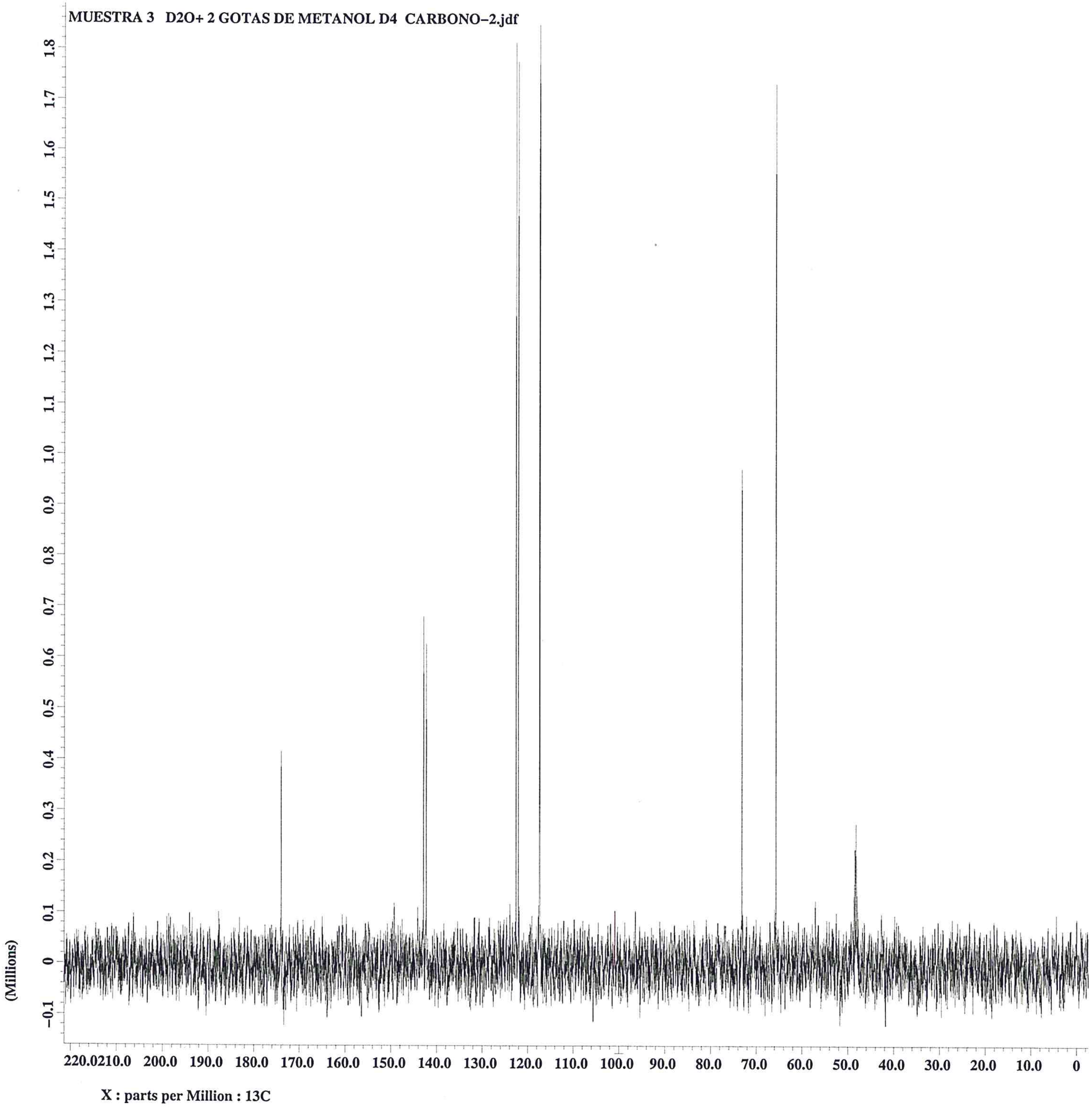


Filename = MUESTRA 3 D2O+ 2 GO
Experiment = single_pulse_dec
Sample_id = I3_030_009
Solvent = D2O
Creation_time = 3-DEC-2014 16:20:47
Author = DELTA
Content = Single Pulse with Bro
Current_time = 4-DEC-2014 12:51:15
Data_format = 1D COMPLEX
Dim_size = 32768
Dim_title = 13C
Dim_units = [ppm]
Dimensions = X
Machine = eclipse2
Revision_time = 3-DEC-2014 17:11:08
Site = Eclipse+ 400
Spectrometer = DELTA_NMR

Field_strength = 9.389766[T] (400[MHz]
X_acq_duration = 1.3008896[s]
X_domain = 13C
X_freq = 100.52530333[MHz]
X_offset = 100[ppm]
X_points = 32768
X_prescans = 4
X_resolution = 0.76870474[Hz]
X_sweep = 25.18891688[kHz]
Irr_domain = 1H
Irr_freq = 399.78219838[MHz]
Irr_offset = 5[ppm]
Clipped = FALSE
Mod_return = 1
Scans = 195
Total_scans = 195
X_90_width = 10.3[us]
X_acq_time = 1.3008896[s]
X_angle = 30[deg]
X_pulse = 3.43333333[us]
Initial_wait = 1[s]
Phase_preset = 3[us]
Recvr_gain = 27
Relaxation_delay = 1[s]
Temp_get = 24.2[dC]
Unblank_time = 2[us]

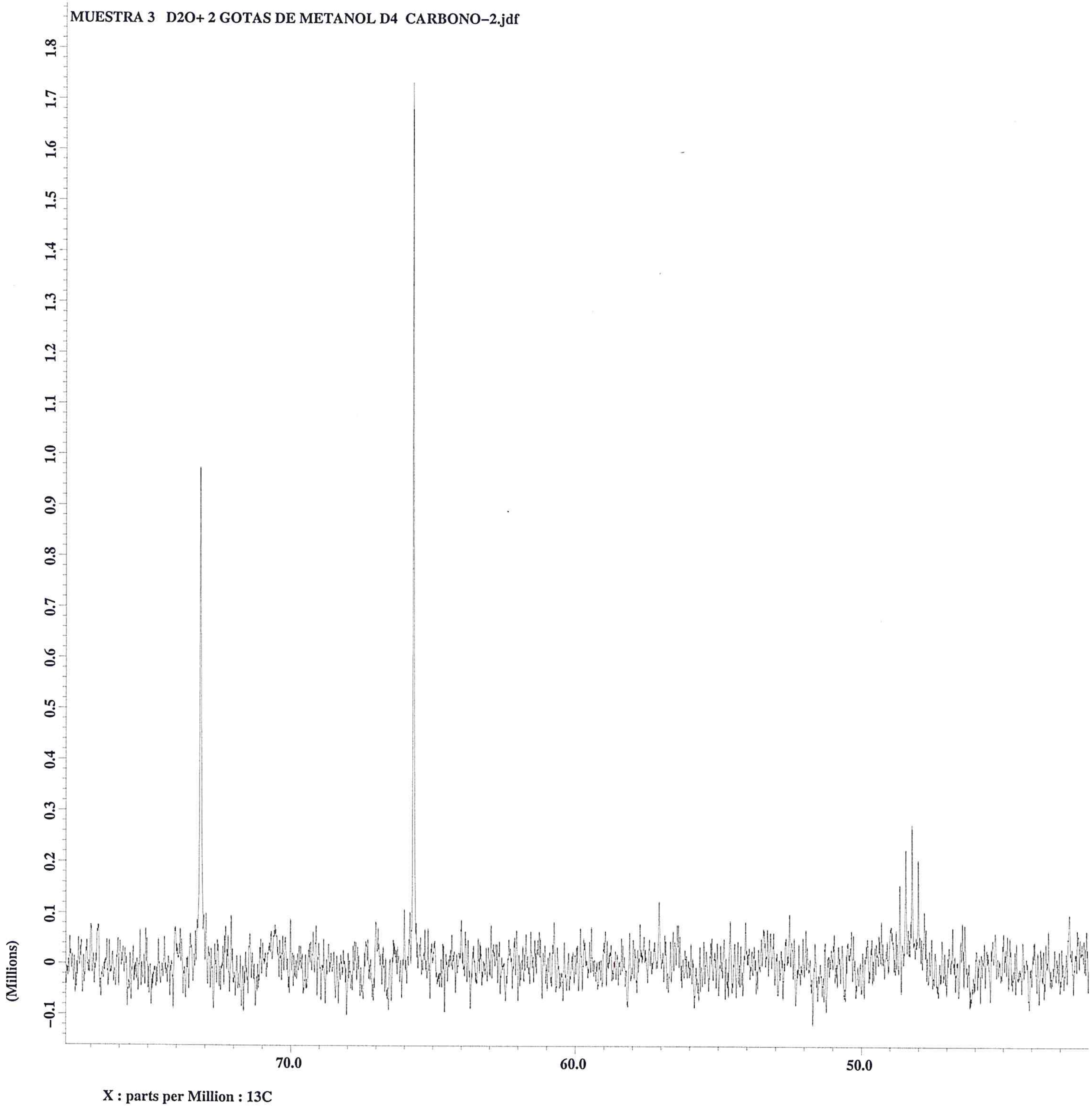


```
Filename          = MUESTRA 3   D2O+ 2 GO
Experiment        = single_pulse_dec
Sample_id         = I3_030_009
Solvent           = D2O
Creation_time     =  3-DEC-2014 16:20:47
Author            = DELTA
Content           = Single Pulse with Bro
Current_time      =  4-DEC-2014 12:53:10
Data_format       = 1D COMPLEX
Dim_size          = 32768
Dim_title         = 13C
Dim_units         = [ppm]
Dimensions        = X
Machine           = eclipse2
Revision_time     =  3-DEC-2014 17:11:08
Site              = Eclipse+ 400
Spectrometer      = DELTA_NMR

Field_strength    = 9.389766[T] (400[MHz]
X_acq_duration    = 1.3008896[s]
X_domain          = 13C
X_freq            = 100.52530333[MHz]
X_offset          = 100[ppm]
X_points          = 32768
X_prescans        = 4
X_resolution      = 0.76870474[Hz]
X_sweep           = 25.18891688[kHz]
Irr_domain        = 1H
Irr_freq          = 399.78219838[MHz]
Irr_offset        = 5[ppm]
Clipped           = FALSE
Mod_return        = 1
Scans             = 195
Total_scans       = 195
X_90_width        = 10.3[us]
X_acq_time        = 1.3008896[s]
X_angle           = 30[deg]
X_pulse           = 3.43333333[us]
Initial_wait      = 1[s]
Phase_preset      = 3[us]
Recvr_gain        = 27
Relaxation_delay  = 1[s]
Temp_get          = 24.2[dC]
Unblank_time      = 2[us]
```

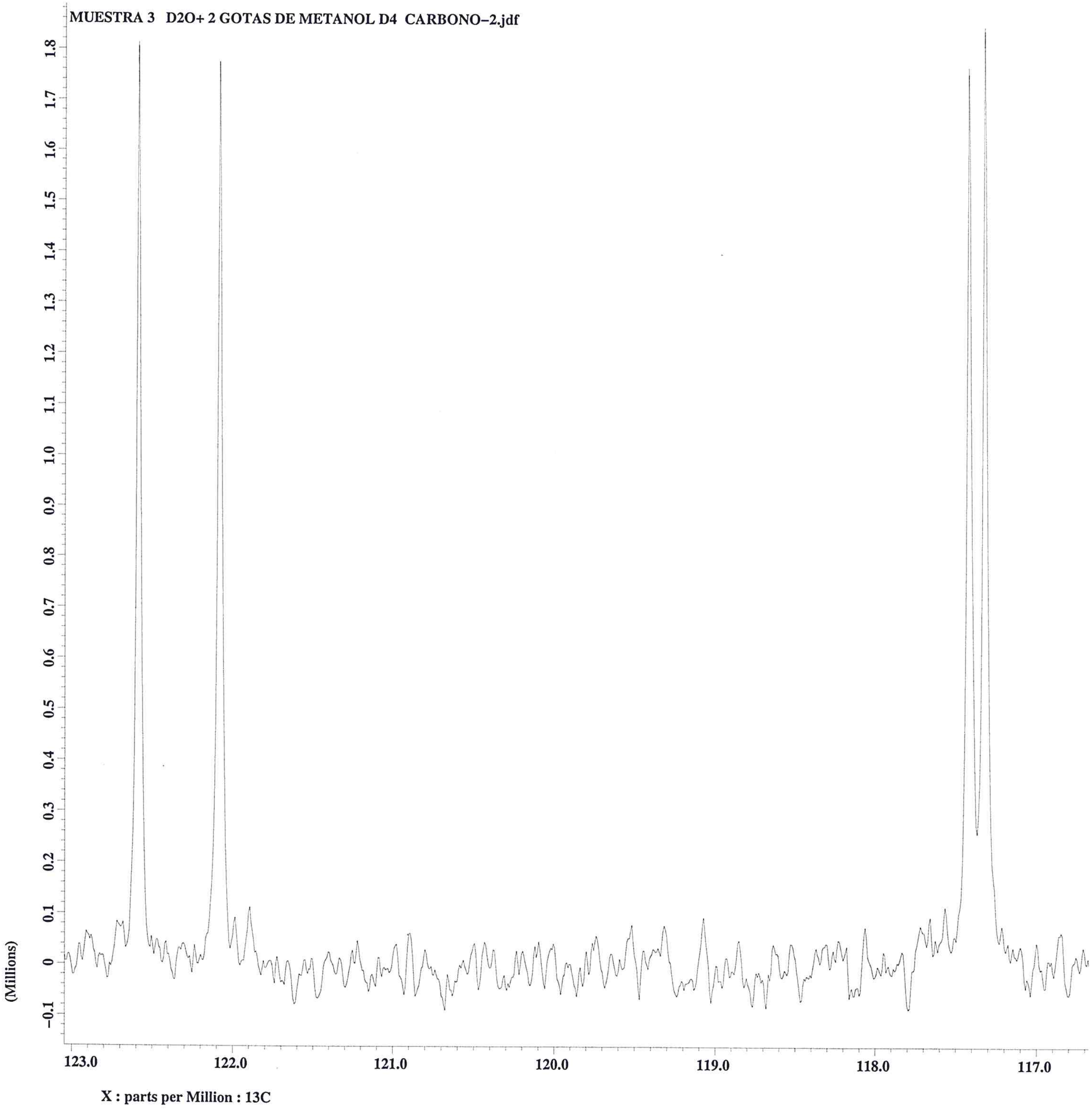


```
Filename           = MUESTRA 3   D2O+ 2 GO
Experiment         = single_pulse_dec
Sample_id          = I3_030_009
Solvent            = D2O
Creation_time      =  3-DEC-2014 16:20:47
Author             = DELTA
Content            = Single Pulse with Bro
Current_time       =  4-DEC-2014 12:53:30
Data_format        = 1D COMPLEX
Dim_size           = 32768
Dim_title          = 13C
Dim_units          = [ppm]
Dimensions         = X
Machine            = eclipse2
Revision_time      =  3-DEC-2014 17:11:08
Site               = Eclipse+ 400
Spectrometer       = DELTA_NMR

Field_strength     = 9.389766[T] (400[MHz]
X_acq_duration     = 1.3008896[s]
X_domain           = 13C
X_freq             = 100.52530333[MHz]
X_offset           = 100[ppm]
X_points           = 32768
X_prescans         = 4
X_resolution       = 0.76870474[Hz]
X_sweep            = 25.18891688[kHz]
Irr_domain         = 1H
Irr_freq           = 399.78219838[MHz]
Irr_offset         = 5[ppm]
Clipped            = FALSE
Mod_return         = 1
Scans              = 195
Total_scans        = 195
X_90_width         = 10.3[us]
X_acq_time         = 1.3008896[s]
X_angle            = 30[deg]
X_pulse            = 3.43333333[us]
Initial_wait       = 1[s]
Phase_preset       = 3[us]
Recvr_gain         = 27
Relaxation_delay   = 1[s]
Temp_get           = 24.2[dC]
Unblank_time       = 2[us]
```

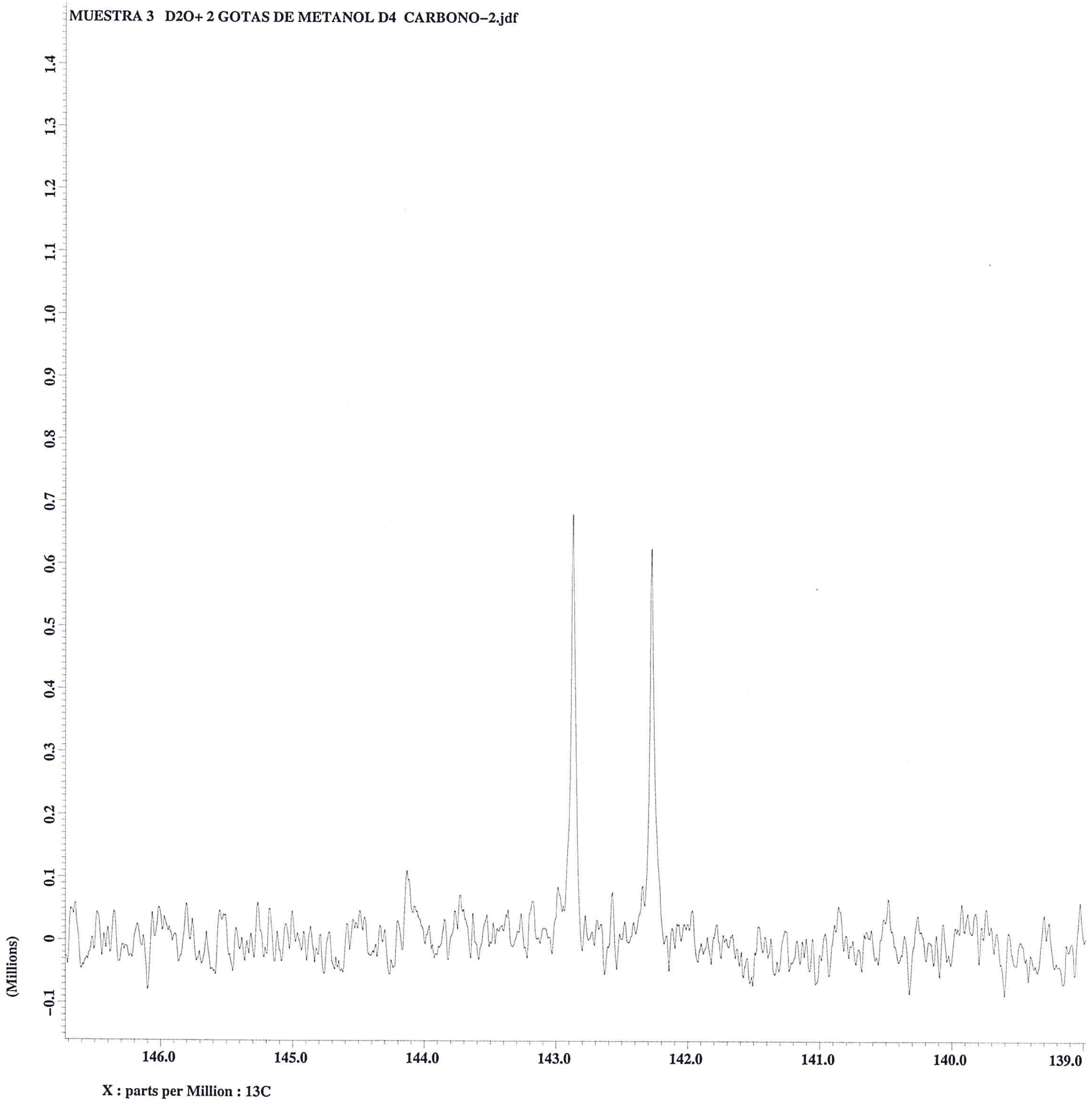


```
Filename          = MUESTRA 3   D2O+ 2 GO
Experiment        = single_pulse_dec
Sample_id         = I3_030_009
Solvent           = D2O
Creation_time     =  3-DEC-2014 16:20:47
Author            = DELTA
Content           = Single Pulse with Bro
Current_time      =  4-DEC-2014 12:53:39
Data_format       = 1D COMPLEX
Dim_size          = 32768
Dim_title         = 13C
Dim_units         = [ppm]
Dimensions        = X
Machine           = eclipse2
Revision_time     =  3-DEC-2014 17:11:08
Site              = Eclipse+ 400
Spectrometer      = DELTA_NMR

Field_strength    = 9.389766[T] (400[MHz]
X_acq_duration    = 1.3008896[s]
X_domain          = 13C
X_freq            = 100.52530333[MHz]
X_offset          = 100[ppm]
X_points          = 32768
X_prescans        = 4
X_resolution      = 0.76870474[Hz]
X_sweep           = 25.18891688[kHz]
Irr_domain        = 1H
Irr_freq          = 399.78219838[MHz]
Irr_offset        = 5[ppm]
Clipped           = FALSE
Mod_return        = 1
Scans             = 195
Total_scans       = 195
X_90_width        = 10.3[us]
X_acq_time        = 1.3008896[s]
X_angle           = 30[deg]
X_pulse           = 3.43333333[us]
Initial_wait      = 1[s]
Phase_preset      = 3[us]
Recvr_gain        = 27
Relaxation_delay  = 1[s]
Temp_get          = 24.2[dC]
Unblank_time      = 2[us]
```

MUESTRA 3 D2O+ 2 GOTAS DE METANOL D4 CARBONO-2.jdf

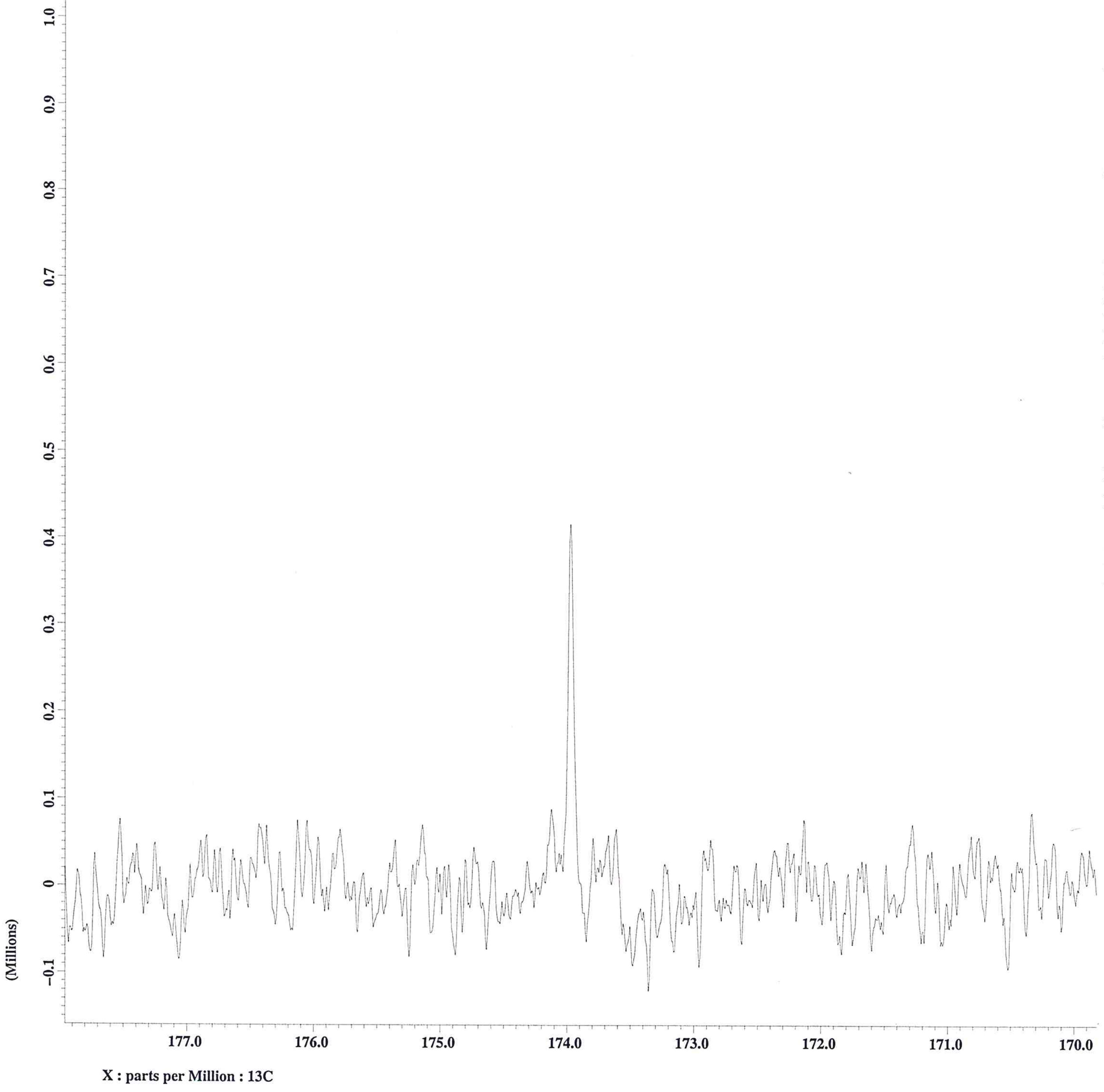


```
Filename         = MUESTRA 3   D2O+ 2 GO
Experiment       = single_pulse_dec
Sample_id        = I3_030_009
Solvent          = D2O
Creation_time    =  3-DEC-2014 16:20:47
Author           = DELTA
Content          = Single Pulse with Bro
Current_time     =  4-DEC-2014 12:53:51
Data_format      = 1D COMPLEX
Dim_size         = 32768
Dim_title        = 13C
Dim_units        = [ppm]
Dimensions       = X
Machine          = eclipse2
Revision_time    =  3-DEC-2014 17:11:08
Site             = Eclipse+ 400
Spectrometer     = DELTA_NMR

Field_strength   = 9.389766[T] (400[MHz]
X_acq_duration   = 1.3008896[s]
X_domain         = 13C
X_freq           = 100.52530333[MHz]
X_offset         = 100[ppm]
X_points         = 32768
X_prescans       = 4
X_resolution     = 0.76870474[Hz]
X_sweep          = 25.18891688[kHz]
Irr_domain       = 1H
Irr_freq         = 399.78219838[MHz]
Irr_offset       = 5[ppm]
Clipped          = FALSE
Mod_return       = 1
Scans            = 195
Total_scans      = 195
X_90_width       = 10.3[us]
X_acq_time       = 1.3008896[s]
X_angle          = 30[deg]
X_pulse          = 3.43333333[us]
Initial_wait     = 1[s]
Phase_preset     = 3[us]
Recvr_gain       = 27
Relaxation_delay = 1[s]
Temp_get         = 24.2[dC]
Unblank_time     = 2[us]
```
